# Physical characteristics of players within the Australian Football League participation pathways: a systematic review

**DOI:** 10.1186/s40798-017-0109-9

**Published:** 2017-12-19

**Authors:** Jade A. Z. Haycraft, Stephanie Kovalchik, David B. Pyne, Sam Robertson

**Affiliations:** 1 Institute of Sport, Exercise and Active Living (ISEAL), Victoria University, P.O. Box 14428, Melbourne, VIC 8001 Australia; 2 Australian Institute of Sport, Canberra, Australia; 3 Research Institute for Sport and Exercise (UCRISE), University of Canberra, Canberra, Australia

**Keywords:** Australian rules football, Physical performance, Talent identification, Sport development pathway

## Abstract

**Background:**

Australian football (AF) players require endurance, strength, speed, and agility to be successful. Tests assessing physical characteristics are commonly used for talent identification; however, their ability to differentiate between players across the Australian Football League’s (AFL) participation pathway remains unclear. The objective of this review was to quantify the physical characteristics of male AF players across the AFL participation pathway.

**Methods:**

A search of databases was undertaken. Studies examining tests of physical performance were included, with 27 meeting the inclusion/exclusion criteria. Study appraisal was conducted using a checklist of selection criteria.

**Results:**

The 20-m sprint time was the most reported test, followed by vertical jump (VJ), AFL planned agility, and 20-m multi-stage fitness test (MSFT). The fastest times for 20-m sprint were for Elite AFL players (range 2.94–3.13 s), with local-level players the slowest (3.22–4.06 s). State Junior Under (U) 18s (58–66 cm) had higher jumps than senior players, with the lowest jumps reported for Local U10s (mean 31 cm). No elite-level data were reported for the AFL planned agility or 20-m MSFT. AFL planned agility times were only reported for talent pathway levels, with large performance variability evident across all levels (8.17–9.12 s). Only mean 20-m MSFT scores were reported from Local U10s to National Draft Camp (6.10–13.50 shuttles).

**Conclusions:**

Talent pathway players exhibit similar mean test scores irrespective of the physical test, with the exception of 20-m sprint and VJ. Physical tests can discriminate between local participation level players but are less useful within the AFL talent pathway.

## Key points

Players forming the AFL talent pathway performed better in all physical tests than players within the AFL local participation pathway.

Players within the AFL talent pathway demonstrate similar physical performances across junior talent levels irrespective of the physical test, with the exception of 20-m sprint and vertical jump tests.

Physical tests will more effectively discriminate levels of competition between AFL local participation pathway players but are less useful within the AFL talent pathway.

## Background

Australian football (AF) is a popular team sport in Australia, with selection of players across the participation pathway partially based on physical characteristics and subjective evaluation of playing ability [1]. Game motion analyses indicate that AF is an intermittent team sport characterised by both high-intensity (high-speed running, sprinting, acceleration, agility) and low-intensity activities (standing, walking, jogging) [2–8]. A player’s ability to progress through to and perform at the elite level requires high levels of aerobic endurance, speed, strength, power, and agility [8].

The physical performance and anthropometric characteristics of AF players have been well documented, with common physical assessments including sprinting, vertical jumps, agility, and multi-stage fitness tests (MSFT) [9–13]. These tests also form part of the annual Australian Football League (AFL) National Draft Combine, where players are evaluated prior to the National Draft. Small-to-moderate (*r* = 0.27–0.31) positive relationships between physical fitness and career progression have been reported in various AF player cohorts [9]. These physical assessments have been primarily conducted not only to inform the selection of players for professional contracts and specific positions, but also to elucidate longitudinal recruiting trends [14].

**Fig. 1:**
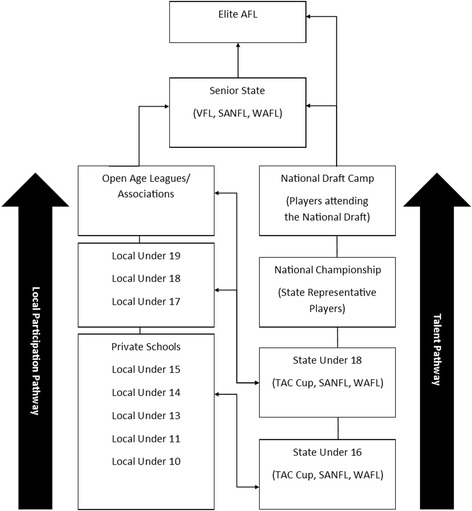
The AFL participation pathway adapted from AFL Development [42, 43]. AFL Australian Football League, TAC Transport Accident Commission (Victorian State Junior Football League), VFL Victorian Football League, SANFL South Australian National Football League, WAFL Western Australian Football League

A review of AF physical performance studies identified a variety of physical test outcomes for players from senior elite, national junior, and state junior levels of AF competition [8]. However, to date, the magnitude of differences in physical performance characteristics along the AFL participation pathway (Fig. 1) has not been reported. Given the prevalence of test use for talent identification and player physical development within the AFL pathway, a review of the relevant literature would help inform recruitment practices [8]. Furthermore, a variety of speed [15–22], agility [12, 23, 24], power [25–31], strength [25, 32–35], movement quality [22, 36–38], and aerobic [15, 39–41] tests have been analysed using AF player samples; however, these tests are not administered using the standardised AFL National Combine protocols. With a large number of studies reporting physical performance measures of AF players across the AFL participation pathway, a review of relevant studies is needed to provide an overview of players’ physical characteristics. Furthermore, a detailed analysis of physical performance measures would provide team coaches and support staff (i.e. strength and conditioning coaches and sport science advisors) benchmarks to inform the physical preparation of players at each level of the AFL participation pathway.

The current AFL participation pathway (Fig. 1) involves two streams that funnel athletes into state and elite senior competitions: the *local participation* pathway and the *talent* pathway [42]. Generally, the majority of players transition through the state-based local participation pathway via the following teams: school/clubs/community (5–11 years of age), junior schools/clubs (12–14 years), youth schools/clubs (15–18 years), to open age league/associations (> 18 years) [43]. The talent pathway runs parallel to the local participation pathway, with a smaller cohort of more elite junior players selected for talent pathway squads based on their objective test outcomes and subjective match performance assessments conducted by coaches and talent scouts [44]. The talent pathway is a national program consisting of regional development squads where talented players can transition through to Under (U) 14–16s state championship teams and national U16s and U18s championship teams [42]. Furthermore, talented AF players may be selected for AFL state academies, sporting centres of excellence, and the National AFL academy [10, 42–44]. Players are selected into senior state AF competitions from either the local participation or talent pathways, with elite players primarily selected through the annual AFL National Draft [9]. While the structure of the AFL participation pathway may provide clear local participation and talent pathways for players, no studies have assessed the physical differences between the levels within both pathways using standardised testing methods.

The aim of this article was to conduct a systematic review of the physical test performance of AF players and establish a comprehensive model of differences in physical performance along the AFL local and talent pathways that informs talent selection, recruitment, and fitness program design.

## Methods

### Design

**Fig. 2:**
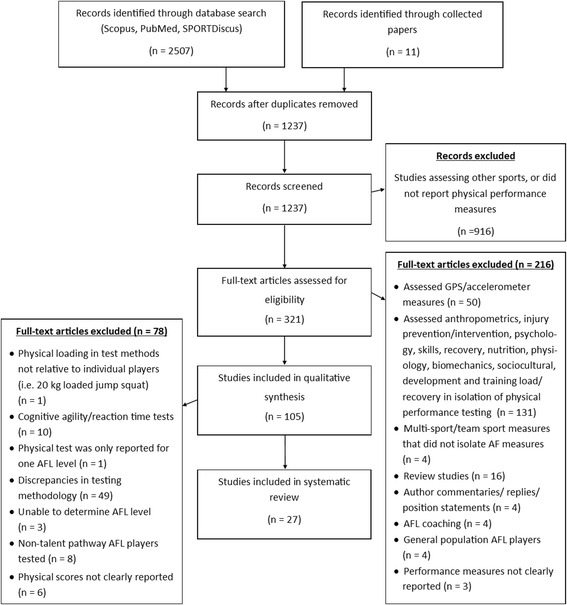
PRISMA flow diagram of study search strategy for systematic review of AFL development pathway. AF Australian Football, AFL Australian Football League, GPS Global Positioning System

The PRISMA (Preferred Reporting Items for Systematic Reviews and Meta-Analyses) statement was used for this systematic review. The PRISMA allows for improved quality of reporting and evaluation of literature for systematic reviews [45]. Studies investigating physical performance tests for speed, change of direction (COD), power, strength, aerobic, anaerobic capacity, and movement quality of male AF players were assessed for potential inclusion. A detailed outline of the search strategy, and criteria used for inclusion/exclusion of studies for review, is shown in Fig. 2.

### Search strategy

A literature search was conducted between August 2015 and March 2017 using SPORTDiscus, PubMed, and Scopus. Key search terms utilised in the search were multiple combinations of AND/OR phrases that included ‘Australian’, ‘football’, ‘physical’, ‘performance’, and ‘talent’. Studies were also identified by examination of citations listed in the collected publications [45].

### Inclusion criteria

The initial search revealed multiple studies as far back as 1970 that investigated physical performance measures of football players. However, no studies prior to 1999 met the inclusion criteria (below) for this review. The final search process specified articles published between 1999 and March 2017. Inclusion criteria for physical performance tests of AF players were as follows: (i) each study had been peer-reviewed and written in English, (ii) abstracts of articles were available, (iii) articles that reported multiple test results were included where results could be extracted and reported in isolation of other tests, and (iv) the testing methods used to collect physical performance data were outlined in detail by the authors.

### Exclusion criteria

Studies were excluded from this review when (i) no physical performance measures for AF players were reported, (ii) AF-specific data were not clearly identifiable, (iii) the article was a review study or author commentary/reply, (iv) the article was an AF coaching-specific study, or (v) the authors tested AF players who had competed in the local participation pathway open age leagues/associations.

### Data extraction

The author list and publication date were recorded for each study identified during the database search. All articles identified in the search were coded as ‘Yes’ or ‘No’ to identify those meeting, or possibly meeting, the inclusion/exclusion criteria. Specifically, sample size, participant characteristics (age, height, and body mass), reported player level within the AFL participation pathway, whether the inclusion/exclusion criteria were reported, and the methodology of physical tests were assessed. Articles were further excluded from this review based on the characteristics detailed in the PRISMA statement (Fig. 2).

### Analysis

Mean and standard deviation of the physical performance test measures were extracted using a customised Microsoft Excel™ (Microsoft Corporation, Santa Rosa, CA, USA) spreadsheet. All data from each study were extracted by the lead author (JH). The magnitude of differences in testing values between each of the participation levels was summarised and displayed using forest plots. These plots were developed for each physical test and player group reported by studies across the multiple AFL participation pathway levels. Each point in the forest plot displays the mean and 95% confidence interval (CI) for a specific player group. Any group whose mean score was not contained within the range of the CIs for any other group within the same AFL level was deemed an outlier. Plots were produced using the RStudio® statistical computing software version 1.0.136 (RStudio, Boston, MA, USA). A formal meta-analysis was explored but not presented in the report because exploratory analysis indicated substantial between-study heterogeneity evident in the majority of analyses.

## Results

### Overview of studies

The initial search process yielded 2507 articles, 1237 were screened and 321 underwent a detailed review for eligibility. Data was extracted from 27 studies that met the inclusion/exclusion criteria (Fig. 2). This data included all reported performance measures for the following physical tests: anaerobic power, aerobic power, speed, strength, power, and COD. Extracted physical data was further classified into AFL participation pathway levels based on the team levels reported in each study. Player data was obtained from the following AFL participation pathway levels: ‘Elite AFL’, ‘Senior State’, ‘National Draft Camp’, ‘National Championship’, ‘State Junior U18’, ‘Private School’, and Local ‘U19’, ‘U18’, ‘U17’, ‘U15’, ‘U13’, ‘U11’, and ‘U10’.

### Speed

**Fig. 3:**
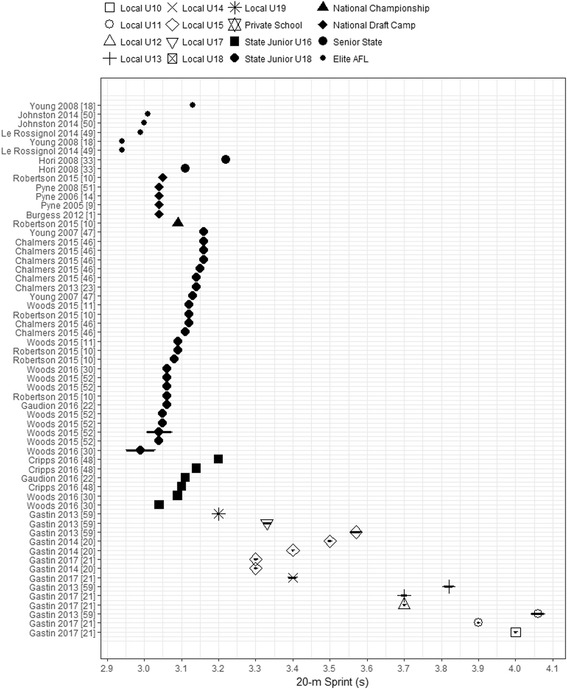
Mean and CIs of reported 20-m sprint times across the AFL participation pathway levels. Values are ordered by position in AFL participation pathway, then by sample size reported for each group. All players’ levels are shown in the legend for consistency though not all levels may have data on the charted performance measure. AFL Australian Football League, U Under

Of the 27 articles reviewed, 20 reported 20-m sprint time across all levels of the AFL participation pathway, with the exception of Private School players. The mean 20-m sprint times across the local participation were observed for the following levels: Local U10 (4.00 s), Local U11 (range 3.90–4.06 s), Local U12 (3.70 s), Local U13 (range 3.70–3.82 s), Local U14 (3.40 s), Local U15 (range 3.30–3.57 s), Local U17 (3.33 s), and Local U19 (3.20 s). Observed mean times were not only slower among the local participation groups, when compared with talent pathway groups, but also more variable (Fig. 3). For example, the difference in the range between means for U15 players was nearly 0.30 s and more than 0.10 s for U13, suggesting greater (variation) inconsistency in sprint performance at the lowest levels of the AFL participation pathway. The slowest observed mean sprint time was reported for the Local U11 group. All mean times from the Local U18 level and below were 3.30 s or slower.

A substantially faster 20-m sprint time is evident as players transition through the AFL participation pathway from Local U10s to Elite AFL competition. The mean range of elite AFL players’ 20-m sprint times was 2.94–3.13 s (Fig. 3). However, one group reported by Young et al. [18] was deemed an outlier within this level, and after removal, the mean range for Elite AFL players was 2.94–3.01 s. The most similar AFL levels in reported 20-m sprint time means and CIs were the State Senior (range 3.11–3.22 s), National Draft Camp (range 3.04–3.05 s), National Championship (mean 3.09 s), State Junior U18s (range 2.99–3.16 s), and State Junior U16s (range 3.04–3.20 s). Multiple studies in these AFL levels had similar 20-m sprint results.

### Change of direction

**Fig. 4:**
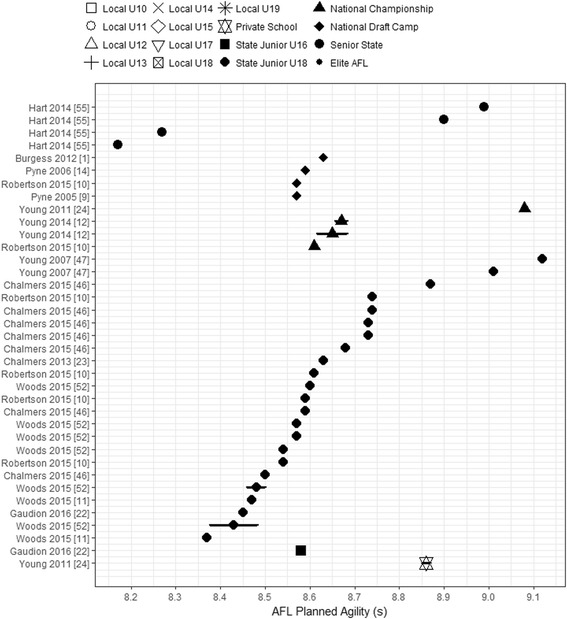
Mean and CIs of reported AFL planned agility times across the AFL participation pathway levels. Values are ordered by position in AFL participation pathway, then by sample size reported for each group. All players’ levels are shown in the legend for consistency though not all levels may have data on the charted performance measure. AFL Australian Football League, U Under

The AFL planned agility time was stated in 13 (48%) of the 27 studies. Only one study reported local participation pathway levels (Private School), with all others reporting talent pathway levels. The Private School players’ mean AFL planned agility time was 8.86 s. Within the talent pathway, no study reported mean AFL planned agility times for Elite AF players, one group reported for State Senior players (range 8.17–8.99 s), four for National Draft Camp (range 8.57–8.63 s), three for National Championship (range 8.61–9.08 s), six reported for State Junior U18 (range 8.37–9.12 s), and one reported for State Junior U16 (8.58 s). The fastest reported AFL planned agility time was recorded for State Senior level players (Fig. 4); however, the range for mean agility time within the four groups was split, with two groups having a mean time range of 8.17–8.27 s and another two ranged between 8.90 and 8.99 s. The slowest reported mean AFL planned agility time was observed for State Junior U18 players (9.12 s) (see Fig. 4). There was a high degree of variability in the State Junior U18 mean times, with three group times for State Junior U18s (Chalmers and Magarey [46] and two by Young and Pryor [47]) above 8.80 s. All other groups reported mean agility times between 8.37 and 8.74 s. The most consistent mean agility times were observed for the National Draft Camp (range 8.57–8.63 s). The National Championship level reported mean agility times (total range 8.61–9.08 s) were consistent across three of the four reported groups (range 8.61–8.67 s), with one outlier observed reported by Young et al. [24]. All groups’ mean agility time and CIs for AFL talent and local participation pathway levels overlapped; as such AFL planned agility performance is similar across these levels.

### Jump tests

**Fig. 5:**
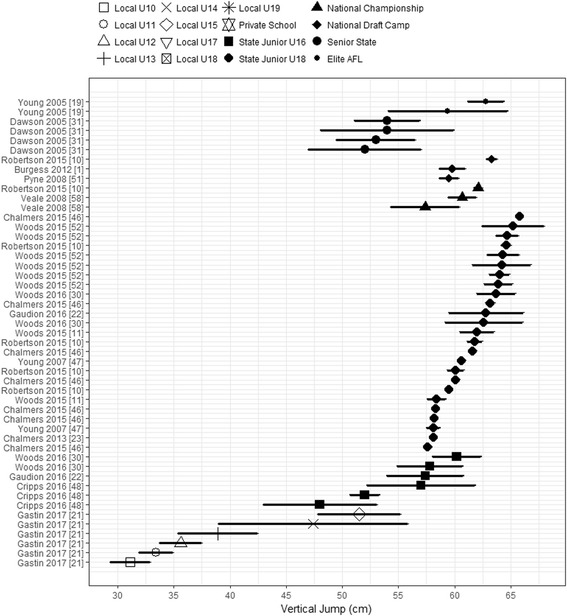
Mean and CIs of reported countermovement jump height across the AFL participation pathway levels. Values are ordered by position in AFL participation pathway, then by sample size reported for each group. All players’ levels are shown in the legend for consistency though not all levels may have data on the charted performance measure. AFL Australian Football League, U Under

Vertical jump performance of AF players was reported in 15 of the 27 reviewed studies (56%), with a number of studies excluded due to variation in testing methods. Only one study reported jump means for Elite and State Senior AF players, respectively, with three studies reporting National Draft level, two studies for National Championship, eight for State Junior U18s, three for State Junior U16s levels, and one for Local U15, U14, U13, U12, U11, and U10s (see Fig. 5).

The lowest reported vertical jump (VJ) means were within the local participation pathway, with a gradual increase in jump height observed with every age competition level increase. The reported jump means within the local participation pathway were as follows: Local U10 (31 cm), Local U11 (33 cm), Local U12 (36 cm), Local U13 (39 cm), Local U14 (47 cm), and Local U15 (52 cm). Overlap in jump means and CIs between each level within the local participation pathway was observed; therefore, similarities in jump performance are assumed between these players.

The highest mean jump height was observed for the State Junior U18 level (range 58–66 cm). The most overlap observed in reported means and CIs was noted in the State Junior U16 (range 48–60 cm), National Championship (range 57–62 cm), National Draft Camp (range 60–63 cm), Senior State (range 52–54 cm), and Elite AFL (range 59–63 cm). Both the National Draft Camp and State Junior U16 groups appeared to have one outlying study (Robertson, Woods and Gastin [10], and Cripps, Hopper and Joyce [48]). When these outliers were removed, the jump mean was 60 cm for National Draft Camp and range 48–52 cm for State Junior U16s. Multiple studies across the AFL talent pathway levels had overlapping CIs, with jump performance observed to be similar among these levels.

No overlap in reported means and CIs was evident for jump height performance between the Local U10s, U11s, and U12s, when compared with all other levels along the AFL participation pathway. High variability in mean jump heights was noted within the Elite AFL, State Senior, State Junior U16s, and Local U10 to U15s as these levels were observed to exhibit the largest CIs.

Another reported measure was the running vertical jump (RVJ) off the left and right foot, with five of the 27 studies reporting running jump performance (see Table 1). Of these, one reported running RVJ scores for State Junior U16s, five for State Junior U18s, one for National Draft Camp, and one for National Championship level players. No running RVJ measures were reported for Elite and Senior State players. As such, no comparison across the entire AFL participation pathway was conducted. The highest running jump score for the right foot was State Junior U18s (mean range 66–75 cm), and for the left (mean range 70–79 cm). The lowest mean scores were recorded for State Junior U16s (right foot 66 cm, left foot 71 cm), with National Draft Camp (right foot 72 cm, left foot 78 cm), and National Championship (right foot 71 cm, left foot 76 cm) players not differing from State Junior U18s. When comparing jump scores for the left and right legs; left leg jumps were higher than those of the right leg for all levels. This trend was found across the State Junior U16s (+ 6 cm), State Junior U18s (+ 4 cm), National Championship (+ 4 cm), and National Draft Camp (+ 6 cm) levels (see Table 1).

**Table 1:** Jump performance measures reported for level of AFL participation pathway. Measures represented as mean ± standard deviation

Study	AFL pathway level	Sample (*n*)	Running vertical jump right (cm)	Running vertical jump left (cm)
Robertson et al. [10]	National Draft Camp	229	72 ± 9	78 ± 9
Robertson et al. [10]	National Championship	219	71 ± 9	76 ± 8
Chalmers and Magarey [46]	State Junior U18	247	67 ± 8	71 ± 8
		219	67 ± 8	70 ± 8
		240	66 ± 9	10 ± 8
		240	69 ± 8	72 ± 8
		220	69 ± 8	73 ± 8
		248	71 ± 8	75 ± 8
		300	74 ± 8	78 ± 8
Chalmers et al. [23]	State Junior U18	382	66 ± 8	71 ± 8
Woods et al. [52]	State Junior U18	212	72 ± 10	79 ± 11
			73 ± 8	78 ± 8
			75 ± 9	79 ± 9
			71 ± 10	77 ± 9
			73 ± 9	78 ± 9
			73 ± 9	78 ± 9
Robertson et al. [10]	State Junior U18	3708	67 ± 9	72 ± 9
	State Junior U18	115	70 ± 8	74 ± 8
	State Junior U18	219	73 ± 9	79 ± 9
	State Junior U18	115	71 ± 8	75 ± 8
Gaudion et al. [22]	State Junior U18	37	73 ± 9	75 ± 8
Gaudion et al. [22]	State Junior U16	40	66 ± 6	71 ± 6

*AFL* Australian Football League, *U* Under

### Aerobic

**Fig. 6:**
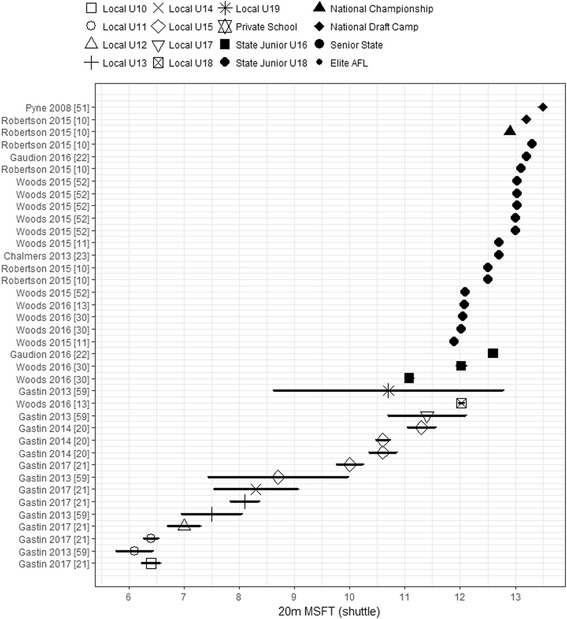
Mean and CIs of 20-m MSFT shuttles reached by levels across the local participation and talent pathways. Values are ordered by position in AFL participation pathway, then by sample size reported for each group. All players’ levels are shown in the legend for consistency though not all levels may have data on the charted performance measure. AFL Australian Football League, MSFT multi-stage fitness test, U Under

Only 11 articles reviewed reported measures of aerobic fitness using the 20-m MSFT shuttles, with none reporting 20-m MSFT shuttles for Elite and Senior State levels. Mean 20-m MSFT performance was only published for AF players involved in local participation and talent pathway levels (see Fig. 6). Two groups reported the mean number of shuttles achieved for National Draft Camp (range 13.20–13.50 shuttles) and National Championship (12.90 shuttles), seven groups reported for State Junior U18 (range 11.90–13.30 shuttles), two reported State Junior U16 (range 11.08–12.60 shuttles), and four reporting local participation levels’ (Local U10 to U19) player scores (range 6.10–12.02 shuttles).

A linear trend was observed (Fig. 6) for 20-m MSFT shuttles in local participation levels, with performance increasing approximately 1 shuttle per age group from U11s (mean range 6.10–6.40 shuttles) to U17s (mean 11.40 shuttles). This trend plateaued as players entered the AFL talent pathway levels. The U10 level (mean 6.40 shuttles) performed slightly better in the 20-MSFT when compared to U11s, despite being a level lower in the local participation pathway. The highest mean 20-m MSFT shuttle reached was observed for the National Draft Camp group (range 13.20–13.50 shuttles). The State Junior U16 (range 11.08–12.60 shuttles), State Junior U18 (range 11.90–13.30 shuttles), National Championship (12.90 shuttles), and National Draft Camp remained consistent in 20-m MSFT test scores range when comparing between these talent pathway levels. Overlap in reported means and CIs for these levels was evident; however, only 8 out of 17 (47%) of the State Junior U18 groups exhibited overlapping CIs with the national level groups. As such, the 20-m MSFT scores between these talent levels were considered to be most similar. No overlap in reported means and CIs were found between the National Championship and National Draft Camp levels, and all other levels reported within the local participation pathway (mean shuttle range 6.10–12.02 shuttles), with the exception of Local U19s (mean 10.70 shuttles). The greatest variability in 20-m MSFT scores was observed within the Local U15 group (range 8.70–11.30 shuttles) and State Junior U16s when compared to all levels across both the entire local participation and talent pathways.

### Strength

Compared to other physical performance measures, reported strength measures across the AFL participation levels were limited. Bench press was the most reported measure, followed by bench pull and back squat (see Table 2). One group reported mean bench pull 1RM measures for Elite AFL (mean 99 kg), one for Senior State (86 kg), and one for State Junior U18s (78 kg). The differences between mean pull strength for Elite and State Junior U18s 1RM was 21 kg with a 13 kg difference between Elite and Senior State players. Mean bench pull 1RM was 8 kg heavier for Senior State than for State Junior U18s. Bench press 1RM was reported for State Junior U18s (mean 88 kg) and Senior State (mean 97 kg) by one group, with two groups reporting Elite AFL 1RM (mean range 103–114 kg). State Junior U18s bench-pressed 9 kg less than State Senior and a further 21 kg less than Elite AFL players, with State Senior pressing 12 kg less than Elite. A comparison of lower body strength across AFL participation pathway levels was not possible as only two groups reported back squat 1RM for elite players, with no performance measures reported for Senior State or levels within the local participation and talent pathways.

**Table 2:** Reported strength performance measures reported for level of AFL participation pathway. Measures represented as mean ± standard deviation

Study	AFL pathway level	Sample (*n*)	Bench press (kg)	Bench pull (kg)
Bilsborough et al. [25]	Elite AFL	19	109 ± 13.3	98.6 ± 5.2
		27	114 ± 7.2	98.5 ± 10.3
Hrysomallis and Buttifant [72]	Elite AFL	20	108.3 ± 13.3	
			102.8 ± 14.0	
			113.8 ± 10.5	
Bilsborough et al. [25]	Senior State	22	96.5 ± 16.6	85.8 ± 9.2
Bilsborough et al. [25]	State Junior U18	21	87.9 ± 12.7	77.8 ± 9.6

*AFL* Australian Football League, *U* Under

### Repeat sprint ability

Studies assessing repeat sprint ability were limited, with only two articles of the 27 reporting repeated sprint times in AF players (see Table 3). The only tests reported across the AFL participation pathway were the 6 × 20 m sprint on 30 s (2 studies), the 6 × 30 m sprint on 20 s (3 studies), and the 6 × 40 m sprint on 15-s test protocols (2 studies). Reported 6 × 20 m sprint times were not different between Elite AFL and Senior State groups, with no studies reporting times for talent or local participation levels. Two groups reported mean total sprint time (s) for 6 × 20 m sprints on 30 s for Elite and Senior State players (range 17.99–19.08 s), with no substantial difference observed. Repeat sprint times were reported for Elite and National Draft Camp level players for the 6 × 30 m sprints on 20 s, with National Draft Camp players’ mean total sprint time approximately 0.50 s slower than that of elite players. Two groups reported measures for Elite and Senior State players using the 6 × 40 m sprints on 15 s, with total sprint time similar between these levels (mean range 32.40–37.00 s).

**Table 3:** Repeat sprint performance measures reported for level of AFL participation pathway. Measures represented as mean ± standard deviation

Study	AFL pathway level	Sample (*n*)	Repeat sprint ability (s)
Aughey [39]	Elite AFL	35	6 × 20 m sprint on 30 s
			18.25 ± 0.26
Elias et al. [77]	Elite AFL	14	6 × 20 m sprint on 30 s
			18.53 ± 0.38
			18.62 ± 0.46
			18.63 ± 0.45
Gastin et al. [15]	Elite AFL	25	6 × 40 m on 15 s
			35.56 ± 0.91
Gastin et al. [16]	Elite AFL	69	6 × 40 m on 15 s
			35.6 ± 1.4
Le Rossignol et al. [49]	Elite AFL	20	6 × 30 m
			25.26 ± 0.55
			25.92 ± 0.80
Aughey [39]	Senior State	35	6 × 20 m sprint on 30 s
			18.27 ± 0.27
Gastin et al. [16]	Senior State	69	6 × 40 m on 15 s
			35.6 ± 1.4
Pyne et al. [51]	National Draft Camp	60	6 × 30 m on 20 s
			25.83 ± 0.6
Gaudion et al. [22]	State Junior U18	37	6 × 30 m on 20 s
			26.89 ± 0.98
Gaudion et al. [22]	State Junior U16	40	6 × 30 m on 20 s
			27.64 ± 0.81

*AFL* Australian Football League, *U* Under

### Movement quality

Movement quality was measured using three different assessments: the Athletic Abilities Assessment (AAA) (1 study), a modified AAA (2 studies), and the Functional Movement Screen (FMS) (1 study) (Table 4). Four groups reported movement ability across the AFL participation pathway, with one group reporting for Elite AFL, three for State Junior U18, one for State Junior U16, and one for Local U18 levels, with no other levels reported. The AAA and modified AAA tests use the same movement assessment criteria, with the exception of the chin-up and total AAA score. The scores for the overhead squat, double lunge, single-leg Romanian deadlift, and push-up were compared across multiple AFL participation pathway levels. Elite AFL players performed better on all AAA and modified AAA exercises (mean score ranges: overhead squat 6–9, double lunge 7–9, single-leg Romanian deadlift 6–9, and push-up 8–9). No substantial differences were noted between State Junior U18s, State Junior U16s, and Local U18s for all exercises in the modified AAA (mean score ranges: overhead squat 3–9, double lunge 3–7, single-leg Romanian deadlift 3–7, and push-up 4–9). State Junior U16 and Local U18 scored approximately 1–2 points lower on all modified AAA exercises than Elite AFL players. The AAA was only reported for Elite AFL and State Junior U18 players, with Elite AFL reported to score slightly higher for the chin-up (6–9 points) and total AAA score (45–63 points) than State U18s (chin-up 4–6; total AAA score 37–47). Comparisons of the AAA performance across the AFL participation pathway were not possible as no other AFL levels were reported. Only one group reported the FMS, with State Junior U18s the only level reported. The mean range for the FMS score was 10.9–15.5 out of a possible 21, with no comparison between AFL levels conducted.

**Table 4:** Movement ability measures reported for level of AFL participation pathway. Measures represented as mean ± standard deviation

Study	AFL pathway level	Sample (*n*)	Overhead squat (score/9)	Double lunge (score/9)	Single-leg Romanian deadlift (score/9)	Push-up (score/9)	Chin-up (score/9)	Total AAA (score/63)	FMS (score/21)
Woods et al. [38]	Elite AFL	*n* = 20	7.5 ± 1.3	Left 7.8 ± 0.9 Right 7.7 ± 1.0	Left 7.5 ± 1.5 Right 7.3 ± 1.4	9.0 ± 0.0	7.8 ± 1.6	53.2 ± 8.5	
Woods et al. [38]	Elite AFL	*n* = 14	7.5 ± 1.6	Left 8.0 ± 1.2 Right 8.1 ± 1.1	Left 7.8 ± 1.02 Right 7.8 ± 1.2	8.7 ± 0.8	8.9 ± 0.2	55.7 ± 7.4	
Woods et al. [38]	State Junior U18	*n* = 13	7.0 ± 1.5	Left 5.8 ± 1.2 Right 5.9 ± 1.1	Left 5.3 ± 1.9 Right 5.0 ± 1.2	7.6 ± 0.9	4.7 ± 1.0	41.6 ± 5.1	
Woods et al. [37]	State Junior U18	*n* = 25	5.2 ± 1.7	Left 5.5 ± 1.0 Right 5.7 ± 0.9	Left 4.8 ± 1.1 Right 4.8 ± 1.1	6.3 ± 0.9			
Gaudion et al. [22]	State Junior U18	*n* = 37	5.1 ± 1.2	Left 5.8 ± 1.0 Right 5.7 ± 0.9	Left 4.1 ± 1.4 Right 4.2 ± 1.4	5.5 ± 1.1			
Chalmers et al. [36]	State Junior U18	*n* = 237							13.2 ± 2.3
Gaudion et al. [22]	State Junior U16	*n* = 40	5.4 ± 1.1	Left 5.6 ± 1.0 Right 5.7 ± 0.9	Left 3.8 ± 1.3 Right 3.8 ± 1.1	4.9 ± 1.2			
Woods et al. [37]	Local U18	*n* = 25	4.0 ± 0.5	Left 4.4 ± 1.4 Right 4.6 ± 1.1	Left 4.1 ± 1.2 Right 4.2 ± 1.1	6.1 ± 0.8			

*AAA* Athletic Ability Assessment, *AFL* Australian Football League, *FMS* Functional Movement Screen, *U* Under

## Discussion

The overarching aim of this review was to (i) conduct a systematic review of physical test performances measures reported for AF players and (ii) establish differences in physical performance across the AFL participation pathway to inform talent selection, recruitment, and fitness program design. The literature search yielded a relatively small number of articles assessing physical performance measures that used consistent testing methods across multiple studies. Moreover, a large number of articles reporting physical tests in AF players were excluded as testing protocols were not consistent across multiple levels of the AFL local participation and talent pathways. Physical testing of AF players is of particular interest in the identification of talented AF players; however, inconsistency in test protocols is a challenge for researchers and the football community in understanding what is required physically of players as they transition from local to elite competition.

As expected, the fastest reported 20-m sprint time in this review was by the Elite AF players [18, 49, 50]; however, the differences in sprint time between Elite and National Junior players were minimal [1, 9–11, 14, 51]. Junior local level players were consistently slower than Junior National and Elite AFL respectively [10, 11, 21, 23, 46, 47, 52]. This finding is supported by those of Papaiakovou et al. [53] and Dupler et al. [54], where more physically mature players between the ages of 14 and 18 years were faster than less mature athletes [53, 54]. Previous studies have reported that 20-m sprint time is purportedly a discriminating factor between drafted and non-drafted players when combined with their 20-m MSFT score [9]. Additionally, 20-m sprint performance is associated with match outcomes across junior state level competitions and players’ subsequent selection into higher AF competitions [9, 30, 47]. However, only one group in this review reported 20-m sprint time for Senior State, with their sprint times slower than junior national and state level players, despite the higher competition ranking within the AFL participation pathway [33]. Furthermore, few differences in 20-m sprint performance across the state junior and national levels of the AFL talent pathway were observed in this review. This outcome is supported by previous work showing that sprint time did not contribute to predicting whether a state junior player may be selected for a junior national team [11]. It appears that the 20-m sprint time may not be a discriminating physical characteristic between junior talent levels; however, it may contribute to player selection from local participation in the talent pathway, or junior talent levels into elite AF competition.

The AFL planned agility run course is 21.8 m in length including one 180° and four 90° turns for assessing a player’s ability to change direction at AFL talent identification camps [1, 9, 12, 55]. Junior and adult AF players’ agility scores were similar across the AFL participation pathway. This is comparable to previous literature, as the AFL planned agility test did not discriminate between drafted and non-drafted AF players, unless players also performed better in the 20-m MSFT and 20-m sprint [9]. Moreover, it has not been shown to be related with career success of players as a stand-alone measure [1]. AFL planned agility time across the 1999–2004 AFL drafts was largely unchanged, despite increases in AF match speeds and improvements in other combine tests (height and 20-m sprint) [14]. However, small- and medium-sized players were slightly faster (effect size (ES) = 0.64–1.11) than taller players or ruckmen. The ability of the AFL planned agility test to identify talented AF players within a positional group is questionable, but it should be useful in discriminating between different positional groups. Shoe surface friction may have influenced the variability in the AFL planned agility tests, with less friction possibly causing a player to slip during a COD test, decreasing their performance [56]. Of the studies reported in this review, only seven [10–12, 22, 46, 47, 52] of the 13 disclosed the surface used to assess player COD, with all using indoor, wooden surfaces. However, when conducting large-scale fitness testing, it is not feasible to supply footwear to players to control for surface friction [57]. As such, surface friction and footwear is a consideration when analysing any COD testing.

The VJ was the second most commonly assessed physical measure reported. However, visually there was greater spread in VJ results within the Elite and Senior State levels when compared across the AFL participation pathway. Studies by Pyne et al. [9] and Burgess, Naughton, and Hopkins [1] reported that VJ performance did not impact on a player’s success within elite AFL competition. Relative VJ scores can be counterintuitive, as lower jump scores were reported for players that were drafted to an AFL team, debuted in the elite competition, played more elite level games, and had greater career potential and value [9]. This data may support the variation in VJ performance in the Senior State group, as the training and development of adult AF players may be focused on other physical and skill attributes, and not on their jumping ability.

Inconsistency in VJ performance was also evident across the junior talent pathway, with the greatest disparity in VJ scores in National Draft Camp players [58], and for State U16s players [22, 52]. This variability in results may be caused by differences in the physical maturity levels of the players tested, with ages ranging between 16 and 18 years. Players may be at different pubertal stages, with Gastin et al. [59] reporting AF players within this age group spanned across the fourth and fifth pubertal stages of development (outlined by Duke et al. [60]). Similar differences in VJ performance were also noted by Jones et al. [61], who reported that jump performance increased with biological maturity in males (*r* = 0.56). While VJ performance may not contribute directly to a player’s success in the elite AFL completion, it may enhance the success of a player’s selection and transition across the AFL talent pathway. Several groups reported that VJ performance was higher in elite junior AF players (state and national levels) than non-selected players [11, 13, 30, 34, 47]. However, other groups reported that VJ does not significantly contribute to the success of players’ progression through the AFL talent pathway [9, 10]. The similarity between the VJ scores in this review supports the mixed findings indicating that the VJ is not a highly reliable tool for talent identification of AF players.

This review only reported running endurance performance measures of aerobic capacity, with the 20-m MSFT considered a proxy test for measuring aerobic capacity of individuals [62, 63]. As expected, a gradual increase in 20-m MSFT scores occurred as players progressed along the AFL participation pathway. This increase in aerobic performance was also reported by two groups [20, 59], who found a significant increase in 20-m MSFT with player maturity. Furthermore, large positive correlations (*r* = 0.65) were observed between the biological maturity of junior AF players and 20-m MSFT score [59]. This trend is not restricted to AF players, with similar increases reported across general population males of the same ages [61, 64]. Substantial differences in 20-m MSFT scores were not observed between National Championship, State Junior U18s, State Junior U16s, and Senior State players. This outcome contradicts previous observations showing 20-m MSFT scores contributed significantly to differences between junior national and junior state level players [11] and subsequent draft success of players [10]. While no studies reported shuttle levels achieved for Elite AFL players, predicted maximal oxygen uptake (VO_2max_) (58.0 ± 3.2 mL kg^−1^ min^−1^) from the 20-m MSFT had small associations with career progression of Elite AFL players [9]. Furthermore, Elite AFL player’s VO_2max_ range between 51 and 68 mL kg^−1^ min^−1^ [39, 65] when measured using a laboratory-based VO_2max_ treadmill test, with these measures providing a guideline as to the estimated VO_2max_ capacity of Elite AFL players. When comparing local participation level players to players within the talent pathway, there was a larger variability in 20-m MSFT shuttle scores for Local U15 players [59]. The standard deviation of test scores was 3 shuttles for U15, which is almost twofold higher than those of the other groups across the talent pathway levels. This observation is explained partly by variations in biological maturity [59] and pubertal stages [60] of players competing in this age group.

Lower body strength is an underlying physical characteristic that affects force generation, thus influencing both injury prevention and power production in team sport athletes’ [33, 66–69]. Unfortunately, only one group [66] reported Elite AFL 1RM back squat and one [33] reported Senior State 1RM front squat as measures of lower body strength. No 1RM strength measures in any lower body exercise were reported for junior and developing AF players. The absence of strength testing literature may relate to concerns regarding the safety and reliability of 1RM testing in inexperienced athletes; however, the 1RM back squat is a reliable measure provided to players who have had 6–12 months of familiarisation with the exercise [70, 71]. Tackling and fending off opponents during AF game play require upper body strength [25, 72]; however, the upper body strength literature was also limited. A gradual increase in bench press and bench pull measures was noted as players progress through the AFL participation pathway. This trend is likely a result of long-term adaptations to specific resistance training [25, 73], in combination with physical maturation of players [74–76]. Clearly, strength development is important in AF players; however, further research is required to profile lower and upper body strength of AF players across the entire local and junior talent pathways.

Repeat sprint ability is considered to be one of the more critical aspects in AFL performance, as the game requires players to repeatedly chase defensively and sprint to create space offensively [8, 15, 49]. Unfortunately, comparisons of repeat sprint ability between AFL participation pathway levels are not possible given inconsistencies across test protocols. One group found repeat sprint ability using the 6 × 30 m sprints on 20 s protocol was a discriminating performance measure between selected and non-selected elite AF player [49]. This protocol is currently used as a test in the annual AFL Draft Combine [51], yet no other studies have determined the relationship between performance in this test, and a players’ likelihood of being drafted.

Another two repeat sprint protocols, the 6 × 20 m sprint on 30 s and the 6 × 40 m sprint on 15 s, have been used in the literature [15, 16, 39, 77]. Of these, Aughey [39] and Elias et al. [77] only reported 6 × 20 m sprint results as a profiling tool for Elite and Senior State level players, with no analysis conducted on repeat sprint testing as a talent discriminating factor. Similarly, the 6 × 40 m sprint protocol reported by Gastin et al. [15] and Gastin et al. [16] was assessed in relation to its influence on injury risks of Elite AFL players, predicting match performance. Neither study evaluated this repeat sprint test as a tool for talent identification. Furthermore, Gastin et al. [15] noted that 6 × 40 m sprint protocol was not significantly associated with match performance in elite AF players. It appears the repeat sprint test may not be a reliable tool for assessing whether a player will become a successful AF player. Future research should focus on reporting repeated sprint measures using uniform protocols across the AFL participation pathway levels to allow for meaningful comparisons between groups. This is essential as repeated sprint testing is currently included in the annual AFL National Draft Combine physical testing battery to identify elite AF players.

Movement quality is an underpinning quality of sporting performance, with AF players requiring strong foundation movements such as squats, lunges, pushing, pulling, and bracing to be successful in competition [38, 78, 79]. Movement quality is measured using an objectively assessed criterion to determine if dysfunctional patterns are present [78, 79]. Three movement assessments (AAA, modified AAA, and FMS) were reported for AFL players within the Elite AFL, State Junior U18, State Junior U16, and Local U18 levels [22, 36–38]. The AAA and modified AAA allowed movement comparisons across the abovementioned levels for the following exercises: overhead squat, double lunge, single-leg Romanian deadlift, and push-up. Furthermore, the AAA or modified AAA has been used as a talent identification tool across Elite AFL, State Junior U18, State Junior U16, and Local U18 levels, with junior talent players (State Junior U18 and State Junior U16) exhibiting lesser movement ability than Elite AFL [22, 37, 38]. Woods et al. [37] did find significant differences in AAA scores between State Junior U18 and Local U18 players for the overhead squat, double lunge (both legs), and single-leg Romanian deadlift (right leg). Additionally, a significant effect between State Junior U18 and State Junior U16 levels for the single-leg Romanian deadlift (left leg) (ES = 0.24, *p* < 0.05) and push-up (ES = 0.52, *p* < 0.05) was noted [22]. However, no substantial differences were observed between levels in the junior talent pathway when scores were pooled in this review. The FMS is another movement screening reported by Chalmers et al. [36]; however, this group only examined the association between the FMS and injury risk in State Junior U18 level. Players with observed asymmetrical movements were more likely to sustain an injury during an AF season [36]. Targeting asymmetry in junior players may reduce injury risk and improve athletic performance and development potential as players transition through the AFL participation pathways [36, 37]. While movement ability was found to be similar within the AFL talent pathway, the differences observed between Elite AFL and talent pathway players highlight the importance of developing junior players’ movement ability.

The 20-m sprint [18, 49, 50], VJ [19], and 6 × 30 m repeated sprint tests [49] were the only AFL Draft Combine tests reported for Elite AFL players. No elite level data were noted for the AFL planned agility, RVJ, or 20-m MSFT, in spite of these physical performance measures forming the physical component of talent identification [9–14]. The limited number of studies reporting Elite AFL players may suggest that elite clubs place less value on physical performance measures as talent identification tools, or they do not release results of these tests to preserve any competitive advantage. Other studies reported that jump performance [1, 9], 20-m MSFT [9], and AFL planned agility time [1, 14] had small to trivial associations with career progression of Elite AFL players unless combined with performances in other physical tests. Furthermore, repeat sprint [15, 16, 39, 77] and strength [25, 72] tests were reported mainly for Elite AFL players, indicating that elite clubs place more value on developing these physical characteristics in players than on other qualities assessed through the AFL Draft Combine test battery. Physical performance tests were not consistent across the entire AFL participation pathway; as such, a testing battery that can provide valuable insight into physical differences across the AFL participation pathway is required.

## Conclusions

The physical tests reported in this review are currently used to assess physical characteristics of players and their subsequent progress through the AFL participation pathway. Elite AFL data was only reported for the 20-m sprint and VJ, with no other physical test results available. Elite AFL players had the fastest reported means for 20-m sprint time, with local level players the slowest. All other sprint performances were similar across the talent levels (State U16s–Elite AFL), as mean and CIs in sprint time overlapped with each other. For VJ performance, the State U18s had, counterintuitively, greater jump heights than senior level players. The lowest jumps were reported for Local U10s; however, reported means and CIs for VJ heights overlapped across the AFL talent pathway, and thus, VJ performances were largely similar. AFL planned agility times were only available in the talent pathway, with mean performance times similar across all groups. The 20-m MSFT mean scores were only reported from Local U10s to National Draft Camp, with similarities in performance between the AFL levels. Furthermore, a linear improvement was evident within the local participation pathway for 20-m MSFT performance (1 shuttle per level) as players progressed through the levels. This trend was plateaued when players entered the talent pathway. Finally, players forming the talent pathway performed better in all physical tests than local participation players. However, when assessing levels within the talent pathway, players across different levels tended to exhibit similar mean test scores for each physical test. Physical tests will more effectively discriminate levels of competition between local participation AFL players but are less useful within the AFL talent pathway.

## References

[1] Burgess D, Naughton G, Hopkins W. Draft-camp predictors of subsequent career success in the Australian Football League. J Sci Med Sport. 2012; doi:10.1016/j.jsams.2012.01.006. 10.1016/j.jsams.2012.01.006 22710084

[2] Coutts AJ, Quinn J, Hocking J, et al. Match running performance in elite Australian rules football. J Sci Med Sport. 2010; doi:10.1016/j.jsams.2009.09.004. 10.1016/j.jsams.2009.09.004 19853508

[3] Dawson B, Hopkinson R, Appleby B, et al. Player movement patterns and game activities in the Australian Football League. J Sci Med Sport. 2004; doi:10.1016/S1440-2440(04)80023-9. 10.1016/s1440-2440(04)80023-9 15518293

[4] Wisbey B, Montgomery PG, Pyne DB, et al. Quantifying movement demands of AFL football using GPS tracking. J Sci Med Sport. 2010; doi:10.1016/j.jsams.2009.09.002. 10.1016/j.jsams.2009.09.002 19897414

[5] Veale JP, Pearce AJ, Carlson JS (2007). Player movement patterns in an elite junior Australian rules football team: an exploratory study. J Sports Sci Med..

[6] Boyd LJ, Ball K, Aughey RJ (2013). Quantifying external load in Australian football matches and training using accelerometers. Int J Sports Physiol Perform..

[7] Hiscock D, Dawson B, Heasman J (2012). Game movements and player performance in the Australian Football League. Int J Perform Anal Sport.

[8] Gray AJ, Jenkins DG. Match analysis and the physiological demands of Australian football. Sports Med. 2010; doi:10.2165/11531400-000000000-00000. 10.2165/11531400-000000000-00000 20364877

[9] Pyne DB, Gardner AS, Sheehan K, et al. Fitness testing and career progression in AFL football. J Sci Med Sport. 2005; doi:10.1016/S1440-2440(05)80043-X. 10.1016/s1440-2440(05)80043-x 16248473

[10] Robertson S, Woods C, Gastin P. Predicting higher selection in elite junior Australian rules football: the influence of physical performance and anthropometric attributes. J Sci Med Sport. 2015; doi:10.1016/j.jsams.2014.07.019. 10.1016/j.jsams.2014.07.019 25154704

[11] Woods CTE, Raynor AJ, Bruce L, et al. Predicting playing status in junior Australian football using physical and anthropometric parameters. J Sci Med Sport. 2015; doi:10.1016/j.jsams.2014.02.006. 10.1016/j.jsams.2014.02.006 24613146

[12] Young W, Rogers N. Effects of small-sided game and change-of-direction training on reactive agility and change-of-direction speed. J Sports Sci. 2014; doi:10.1080/02640414.2013.823230. 10.1080/02640414.2013.823230 24016360

[13] Woods CT, Raynor AJ, Bruce L, et al. The application of a multi-dimensional assessment approach to talent identification in Australian football. J Sports Sci. 2016; doi:10.1080/02640414.2016.1142668. 10.1080/02640414.2016.1142668 26862858

[14] Pyne DB, Gardner AS, Sheehan K, et al. Positional differences in fitness and anthropometric characteristics in Australian football. J Sci Med Sport. 2006; doi:10.1016/j.jsams.2005.10.001. 10.1016/j.jsams.2005.10.001 16580878

[15] Gastin PB, Fahrner B, Meyer D, et al. Influence of physical fitness, age, experience, and weekly training load on match performance in elite Australian football. J Strength Cond Res. 2013; doi:10.1519/JSC.0b013e318267925f. 10.1519/JSC.0b013e318267925f 22820206

[16] Gastin PB, Meyer D, Huntsman E, et al. Increase in injury risk with low body mass and aerobic-running fitness in elite Australian football. Int J Sports Physiol Perform. 2015; doi:10.1123/ijspp.2014-0257. 10.1123/ijspp.2014-0257 25365588

[17] Young W, Cormack S, Crichton M (2011). Which jump variables should be used to assess explosive leg muscle function?. Int J Sports Physiol Perform..

[18] Young W, Russell A, Burge P (2008). The use of sprint tests for assessment of speed qualities of elite Australian rules footballers. Int J Sports Physiol Perform..

[19] Young WB, Newton RU, Doyle TLA, et al. Physiological and anthropometric characteristics of starters and non-starters and playing positions in elite Australian rules football: a case study. J Sci Med Sport. 2005; doi:10.1016/S1440-2440(05)80044-1. 10.1016/s1440-2440(05)80044-1 16248474

[20] Gastin PB, Bennett G. Late maturers at a performance disadvantage to their more mature peers in junior Australian football. J Sports Sci. 2014; doi:10.1080/02640414.2013.843016. 10.1080/02640414.2013.843016 24073850

[21] Gastin PB, Tangalos C, Torres L, et al. Match running performance and skill execution improves with age but not the number of disposals in young Australian footballers. J Sports Sci. 2017; doi:10.1080/02640414.2016.1271137. 10.1080/02640414.2016.1271137 28054492

[22] Gaudion S, Kenji D, Sinclair W, et al. Identifying the physical fitness, anthropometric and athletic movement qualities discriminant of developmental level in elite junior Australian football: implications for the development of talent. J Strength Cond Res. 2017; doi:10.1519/JSC.0000000000001682. 10.1519/JSC.0000000000001682 27787473

[23] Chalmers S, Magarey ME, Esterman A, et al. The relationship between pre-season fitness testing and injury in elite junior Australian football players. J Sci Med Sport. 2013; doi:10.1016/j.jsams.2012.09.005. 10.1016/j.jsams.2012.09.005 23092650

[24] Young W, Farrow D, Pyne D, et al. Validity and reliability of agility tests in junior Australian football players. J Strength Cond Res. 2011; doi:10.1519/JSC.0b013e318215fa1c. 10.1519/JSC.0b013e318215fa1c 22076089

[25] Bilsborough JC, Greenway KG, Opar DA, et al. Comparison of anthropometry, upper-body strength, and lower-body power characteristics in different levels of Australian football players. J Strength Cond Res. 2015; doi:10.1519/JSC.0000000000000682. 10.1519/JSC.0000000000000682 25226309

[26] Buchheit M, Racinais S, Bilsborough J, et al. Adding heat to the live-high train-low altitude model: a practical insight from professional football. Br J Sports Med. 2013; doi:10.1136/bjsports-2013-092559. 10.1136/bjsports-2013-092559 PMC3903152 24282209

[27] Caia J, Doyle TLA, Benson AC. A cross-sectional lower-body power profile of elite and subelite Australian football players. J Strength Cond Res. 2013; doi:10.1519/JSC.0b013e3182815743. 10.1519/JSC.0b013e3182815743 23302748

[28] Cormack SJ, Newton RU, McGulgan MR (2008). Reliability of measures obtained during single and repeated countermovement jumps. Int J Sports Physiol Perform..

[29] Crow JF, Buttifant D, Kearny SG, et al. Low load exercises targeting the gluteal muscle group acutely enhance explosive power output in elite athletes. J Strength Cond Res. 2012; doi:10.1519/JSC.0b013e318220dfab. 10.1519/JSC.0b013e318220dfab 22233788

[30] Woods CT, Cripps A, Hopper L, et al. A comparison of the physical and anthropometric qualities explanatory of talent in the elite junior Australian football development pathway. J Strength Cond Res. 2016; doi:10.1016/j.jsams.2016.11.002. 10.1016/j.jsams.2016.11.002 27899276

[31] Dawson B, Gow S, Modra S (2005). Effects of immediate post-game recovery procedures on muscle soreness, power and flexiblity levels over the next 48 hours. J Sci Med Sport.

[32] Hart NH, Nimphius S, Spiteri T (2014). Leg strength and lean mass symmetry influences kicking performance in Australian football. J Sports Sci Med.

[33] Hori N, Newton RU, Andrews WA, et al. Does performance of hang power clean differentiate performance of jumping, sprinting, and changing of direction? J Strength Cond Res. 2008; doi:10.1519/JSC.0b013e318166052b. 10.1519/JSC.0b013e318166052b 18550955

[34] Keogh J (1999). The use of physical fitness scores and anthropometric data to predict selection in an elite under 18 Australian rules football team. J Sci Med Sport.

[35] Woods MA, Watsford ML, Cavanagh BP (2015). Factors affecting jump performance in professional Australian rules footballers. J Sports Med Phys Fitness.

[36] Chalmers S, Fuller JT, Debenedictis TA (2017). Asymmetry during preseason functional movement screen testing is associated with injury during a junior Australian football season. J Sci Med Sport.

[37] Woods CT, Banyard HG, McKeown I (2016). Discriminating talent identified junior Australian footballers using a fundamental gross athletic movement assessment. J Sport Sci Med.

[38] Woods CT, McKeown I, Haff GG, et al. Comparison of athletic movement between elite junior and senior Australian football players. J Sport Sci. 2015; doi:10.1080/02640414.2015.1107185. 10.1080/02640414.2015.1107185 26525174

[39] Aughey RJ. Widening margin in activity profile between elite and sub-elite Australian football: a case study. J Sci Med Sport. 2013; doi:10.1016/j.jsams.2012.10.003. 10.1016/j.jsams.2012.10.003 23391433

[40] Bellenger CR, Fuller JT, Nelson MJ, et al. Predicting maximal aerobic speed through set distance time-trials. Eur J Appl Physiol. 2015; doi:10.1007/s00421-015-3233-6. 10.1007/s00421-015-3233-6 26242778

[41] Inness MWH, Billaut F, Aughey RJ (2016). Team-sport athletes’ improvement of performance on the Yo-Yo Intermittent Recovery Test Level 2, but not of time-trial performance, with intermittent hypoxic training. Int J Sports Physiol Perform..

[42] AFL Community. Participation and talent pathways. 2016. http://www.aflcommunityclub.com.au/index.php?id=26. Accessed 10 May 2016.

[43] AFL Community. DraftStar. 2017. http://www.aflcommunityclub.com.au/index.php?id=1771. Accessed 11 Nov 2017.

[44] Burgess D, Naughton G, Norton K (2012). Quantifying the gap between under 18 and senior AFL football: 2003 and 2009. Int J Sports Physiol Perform..

[45] Moher D, Liberati A, Tetzlaff J (2009). Reprint—Preferred Reporting Items for Systematic Reviews and Meta-Analyses: the PRISMA statement. Phys Ther Sport.

[46] Chalmers S, Magarey M (2015). Annual improvement in fitness test performance for elite junior Australian football cohorts. J Sci Med Sport.

[47] Young WB, Pryor L. Relationship between pre-season anthropometric and fitness measures and indicators of playing performance in elite junior Australian rules football. J Sci Med Sport. 2007; doi:10.1016/j.jsams.2006.06.003. 10.1016/j.jsams.2006.06.003 16854624

[48] Cripps AJ, Hopper L, Joyce C. Maturity, physical ability, technical skill and coaches’ perception of semi-elite adolescent Australian footballers. Pediatr Exerc Sci. 2016; doi:10.1123/pes.2015-0238. 10.1123/pes.2015-0238 27046936

[49] Le Rossignol P, Gabbett TJ, Comerford D, et al. Repeated-sprint ability and team selection in Australian Football League players. Int J Sports Physiol Perform. 2014; doi:10.1123/IJSPP.2013-0005. 10.1123/ijspp.2013-0005 23628711

[50] Johnston R, Watsford M, Pine M, et al. Standardisation of acceleration zones in professional field sport athletes. Int J Sports Sci Coach. 2014; doi:10.1260/1747-9541.9.5.1161.

[51] Pyne DB, Saunders PU, Montgomery PG, et al. Relationships between repeated sprint testing, speed, and endurance. J Strength Cond Res. 2008; doi:10.1519/JSC.0b013e318181fe7a. 10.1519/JSC.0b013e318181fe7a 18714221

[52] Woods CT, Robertson SJ, Gastin PB (2015). Does relative age distribution influence the physical and anthropometric profiles of drafted under 18 Australian footballers? An investigation between the 2010 and 2013 seasons. Talent Dev Excellence.

[53] Papaiakovou G, Giannakos A, Michailidis C (2009). The effect of chronological age and gender on the development of sprint performance during childhood and puberty. J Strength Cond Res..

[54] Dupler TL, Amonette WE, Coleman AE, et al. Anthropometric and performance differences among high-school football players. J Strength Cond Res. 2010; doi:10.1519/JSC.0b013e3181e4f9ec. 10.1519/JSC.0b013e3181e4f9ec 20634741

[55] Hart NH, Spiteri T, Lockie RG, et al. Detecting deficits in change of direction performance using the preplanned multidirectional Australian Football League agility test. J Strength Cond Res. 2014; doi:10.1519/jsc.0000000000000587. 10.1519/JSC.0000000000000587 24942167

[56] Damm L, Starbuck C, Stocker N, et al. Shoe-surface friction in tennis: influence on plantar pressure and implications for injury. Footwear Sci. 2014; doi:10.1080/19424280.2014.891659.

[57] Dos' Santos T, Thomas C, Jones PA, et al. Mechanical determinants of faster change of direction speed performance in male athletes. J Strength Cond Res. 2017; doi:10.1519/JSC.0000000000001535. 10.1519/JSC.0000000000001535 27379954

[58] Veale JP, Pearce AJ, Koehn S, et al. Performance and anthropometric characteristics of prospective elite junior Australian footballers: a case study in one junior team. J Sci Med Sport. 2008; doi:10.1016/j.jsams.2006.12.119. 10.1016/j.jsams.2006.12.119 17544327

[59] Gastin PB, Bennett G, Cook J. Biological maturity influences running performance in junior Australian football. J Sci Med Sport. 2013; doi:10.1016/j.jsams.2012.05.005. 10.1016/j.jsams.2012.05.005 22727755

[60] Duke PM, Litt IF, Gross RT (1980). Adolescents’ self-assessment of sexual maturation. Pediatrics.

[61] Jones MA, Hitchen PJ, Stratton G (2000). The importance of considering biological maturity when assessing physical fitness measures in girls and boys aged 10 to 16 years. Ann Hum Biol.

[62] Wagner PD (1996). Determinants of maximal oxygen transport and utilization. Annu Rev Physiol.

[63] Aandstad A, Holme I, Berntsen S (2011). Validity and reliability of the 20 meter shuttle run test in military personnel. Mil Med.

[64] Beets MW, Pitelli KHA (2004). Comparison of shuttle-run performance between midwestern youth and their national and international counterparts. Pediatr Exerc Sci.

[65] Lorenzen C, Williams MD, Turk PS (2009). Relationship between velocity reached at VO_2max_ and time-trial performances in elite Australian rules footballers. Int J Sports Physiol Perform..

[66] Nibali ML, Chapman DW, Robergs RA, et al. A rationale for assessing the lower-body power profile in team sport athletes. J Strength Cond Res. 2013; doi:10.1519/JSC.0b013e3182576feb. 10.1519/JSC.0b013e3182576feb 22505130

[67] Wisløff U, Castagna C, Helgerud J (2004). Strong correlation of maximal squat strength with sprint performance and vertical jump height in elite soccer players. Br J Sports Med.

[68] Scase E, Cook J, Makdissi M, et al. Teaching landing skills in elite junior Australian football: evaluation of an injury prevention strategy. Br J Sports Med. 2006; doi:10.1136/bjsm.2006.025692. 10.1136/bjsm.2006.025692 PMC2465083 16920776

[69] Orchard J, Marsden J, Lord S (1997). Preseason hamstring muscle weakness associated with hamstring muscle injury in Australian footballers. Am J Sports Med.

[70] Comfort P, McMahon JJ (2015). Reliability of maximal back squat and power clean performances in inexperienced athletes. J Strength Cond Res..

[71] Kraemer W, Fry A, Ratamess N, Maud P, Foster C (1995). Strength testing: development and evaluation of methodology. Physiological assessment of human fitness.

[72] Hrysomallis C, Buttifant D (2012). Influence of training years on upper-body strength and power changes during the competitive season for professional Australian rules football players. J Sci Med Sport.

[73] Baker DG. 10-year changes in upper body strength and power in elite professional rugby league players—the effect of training age, stage, and content. J Strength Cond Res. 2013; doi:10.1519/JSC.0b013e318270fc6b. 10.1519/JSC.0b013e318270fc6b 23358318

[74] Philippaerts RM, Vaeyens R, Janssens M (2006). The relationship between peak height velocity and physical performance in youth soccer players. J Sports Sci.

[75] Lloyd RS, Oliver JL, Faigenbaum AD, et al. Chronological age vs. biological maturation: implications for exercise programming in youth. J Strength Cond Res. 2014; doi:10.1519/JSC.0000000000000391. 10.1519/JSC.0000000000000391 24476778

[76] Matthys S, Vaeyens R, Coelho-e-Silva M (2012). The contribution of growth and maturation in the functional capacity and skill performance of male adolescent handball players. Int J Sports Med.

[77] Elias GP, Varley MC, Wyckelsma VL (2012). Effects of water immersion on posttraining recovery in Australian footballers. Int J Sports Physiol Perform.

[78] Cook G, Burton L, Hoogenboom BJ (2014). Functional movement screening: the use of fundamental movements as an assessment of function—part 1. Int J Sports Phys Ther..

[79] McKeown I, Taylor-McKeown K, Woods C (2014). Athletic ability assessment: a movement assessment protocol for athletes. Int J Sports Phys Ther.

